# Reducing Occupational Sitting Time and Improving Worker Health: The Take-a-Stand Project, 2011

**DOI:** 10.5888/pcd9.110323

**Published:** 2012-10-11

**Authors:** Nicolaas P. Pronk, Abigail S. Katz, Marcia Lowry, Jane Rodmyre Payfer

**Affiliations:** Author Affiliations: Abigail S. Katz, HealthPartners, HealthPartners Research Foundation, and JourneyWell, Minneapolis, Minnesota; Marcia Lowry, HealthPartners, Minneapolis, Minnesota; Jane Rodmyre Payfer, Ergotron, Eagan, Minnesota.

## Abstract

**Background:**

Prolonged sitting time is a health risk. We describe a practice-based study designed to reduce prolonged sitting time and improve selected health factors among workers with sedentary jobs.

**Community Context:**

We conducted our study during March–May 2011 in Minneapolis, Minnesota, among employees with sedentary jobs.

**Methods:**

Project implementation occurred over 7 weeks with a baseline period of 1 week (period 1), an intervention period of 4 weeks (period 2), and a postintervention period of 2 weeks (period 3). The intervention group (n = 24) received a sit-stand device during period 2 designed to fit their workstation, and the comparison group (n = 10) did not. We used experience-sampling methods to monitor sitting behavior at work during the 7 weeks of the project. We estimated change scores in sitting time, health risk factors, mood states, and several office behaviors on the basis of survey responses.

**Outcome:**

The Take-a-Stand Project reduced time spent sitting by 224% (66 minutes per day), reduced upper back and neck pain by 54%, and improved mood states. Furthermore, the removal of the device largely negated all observed improvements within 2 weeks.

**Interpretation:**

Our findings suggest that using a sit-stand device at work can reduce sitting time and generate other health benefits for workers.

## Background

Prolonged sitting time (as a specific instance of sedentary behavior), independent of physical activity, has emerged as a risk factor for various negative health outcomes. Study results have demonstrated associations of prolonged sitting time with premature mortality (1–3); chronic diseases such as cardiovascular disease, diabetes, and cancer (4–7); metabolic syndrome (5,6); and obesity (5,7). In contrast, breaks in prolonged sitting time have been correlated with beneficial metabolic profiles among adults, suggesting that frequent breaks in sedentary activity may explain lower health risk related to waist circumference, body mass index (BMI), triglyceride levels, and 2-hour plasma glucose levels (8).

In the contemporary work place, many workers spend more than half of their entire work day seated (9), so the work place represents a community venue for the promotion of physical activity and the reduction of sedentary time (10). Attempts to reduce sedentary behavior at work through the introduction of devices to stimulate breaks in sitting time have shown promise (11–13). However, systematic reviews indicate a lack of evidence of effectiveness related to work-site–based interventions intended to reduce sitting time (14).

## Community Context

**Figure 1:**
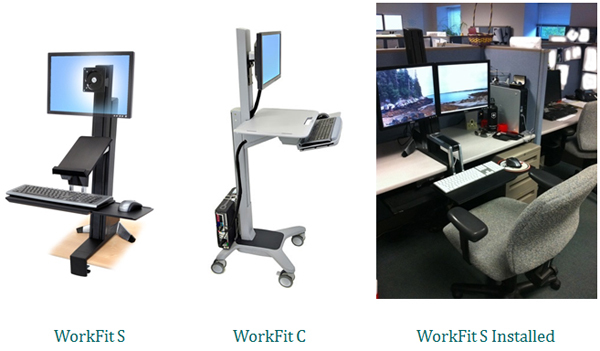
Sit-stand devices used in the Take-a-Stand Project, Minneapolis, Minnesota, 2011.

Approaches to changing behavior in the work place may be directed at the levels of individuals, groups of individuals, organizations, or work environments (10). Ideally, programs are implemented at multiple levels simultaneously because individual efforts at changing behavior tend to more successful if they are complemented by supportive environments (10). Our project considers the physical and psychosocial environments by redesigning work stations to include a sit-stand device for the employee. Figure 1 shows both devices used in this project (WorkFit S or WorkFit C, Ergotron, Inc, Eagan, Minnesota) and an example of a workstation adapted for the WorkFit S. These devices can be elevated or lowered to an appropriate height so that an employee can work either standing or sitting, and employees can change from a standing or seated position at any time during the work day without interrupting their work flow or process. We presented this adaption to the work stations in the context of a comprehensive and multicomponent corporate health and well-being program. This program provides access to physical activity resources, offers incentives to participate in physical activity, and is supported by managerial and supervisory policies and protocols that promote worker health in the work place. Employees could be eligible for financial incentives by being actively engaged in the organization’s health and well-being program as a whole; however, there were no financial incentives to participate in the Take-a-Stand Project itself.

The Take-a-Stand Project was a partnership among a sit-stand device manufacturer, Ergotron, Inc, Eagan, Minnesota; an employer, HealthPartners, a large nonprofit, member-governed health system in Bloomington, Minnesota; and the employees of the Health Promotion Department at HealthPartners in Minneapolis, Minnesota. Both Ergotron and HealthPartners have an interest in improving human performance through ergonomic and health-related solutions (15). Participating employees had the opportunity to comment on their interest in the timing and approach of the project and to share their experiences with the project.

The typical jobs of project participants involved desk work, such as computer operations, telephone interactions, and administrative duties. Participants’ jobs were characterized as sedentary with prolonged periods of sitting time.

The Take-a-Stand Project had 2 specific objectives: to study the effect of a sit-stand device on time spent sitting at work and to assess the effect of reduced sitting time on selected health-related outcomes, mood states, and indices of work performance and office behavior. It was conducted during March–May 2011.

## Methods

### Participants

Potential participants were informed of the project’s objectives, rationale, and protocol during a question-and-answer session. Participation was entirely voluntary. Supervisors and managers actively supported the project’s implementation. Eligible project participants (N = 55) included health promotion department employees with sedentary jobs, including administration, customer service, account management, health coaching, and management. Thirty-four employees signed up to participate, 24 for the intervention group and 10 for the comparison group.

### Project protocol and measures

We used a nonrandomized interrupted time series approach (a study design in which, over time, groups go through different exposures) with a 2-group pre-post comparison design. The project was implemented over a 7-week period with a baseline period of 1 week (period 1) in which all 34 participants were monitored without intervention; an intervention period of 4 weeks during which sit-stand devices were installed for the intervention group only (period 2); and a postintervention period of 2 weeks (period 3) without intervention for either group.

For objective 1, we monitored sitting, standing, and walking behavior for the entire 7-week duration of the project by using experience-sampling methodology (ESM), a methodology that described real-world situations by frequent sampling of a situation or behavior (16). ESM has been used successfully in studies related to various health factors, populations, and settings, including the work place (16,17). We provided all participants a prepaid cellular telephone and sent text messages at 3 random times throughout the course of the work day. Immediately upon receiving a text message, participants responded to the question, “Tell us what you are doing right now: sitting, standing, or walking?” by using 0, 1, or 2 for sitting, standing, or walking, respectively. An average score (range, 0–2, representing sitting to standing to walking) was used as the outcome measure for objective 1 to indicate a reduction in sitting behavior (not sitting time; rather, whether or not participants changed their sitting *behavior* as quantified through the frequency measurement using the ESM). Average scores for periods 1, 2, and 3, respectively, were used to test for differences in sitting behavior between periods. One participant was absent for more than 4 weeks during the study; we deleted that participant’s responses from this part of the analysis.

For objective 2, we used surveys to assess the effect of reduced sitting time on selected health-related outcomes, mood states, and office behaviors. All participants were asked to complete surveys at baseline, at the end of week 5, and at the end of week 7. The survey included questions related to participant characteristics, including age, sex, body weight and height, physical activity levels and intensities, estimated cardiorespiratory fitness (18), self-perceived health status, role physical (degree of problems with work or other daily activities as a result of physical health issues), role emotional (degree of problems with work or other daily activities as a result of emotional health issues), and job type. Outcomes related to objective 2 were measured by using this same survey and included participants’ estimates of time spent sitting per day, ratings of lower back pain and upper back and neck pain, informal face-to-face time with coworkers, time spent in physical activity breaks, and the Profile of Mood States (POMS) questionnaire (19). Validation studies have reported internal consistency (α) coefficients for the POMS subscales ranging from 0.84 to 0.95 and test-retest reliability coefficients ranging from 0.65 to 0.74 (19). The survey conducted at the end of week 7 also included an opportunity for all 34 participants to provide comments and feedback regarding their experiences related to the project (Appendix). The 24 intervention-group participants answered a series of questions about the overall perceived benefits of the sit-stand device.

### Statistical analyses

Participants’ characteristics and outcomes data were compiled for both groups. The ESM data were averaged for each group by period. The differences between periods were assessed by using paired *t* tests. Similar analyses were applied to the survey data for self-reported sitting time, health-related outcomes, office behaviors, and mood states. Statistical significance was considered at *P* < .05, and after the application of a Bonferroni multiple comparison correction, at *P* < .017. Finally, Pearson’s correlation coefficients were calculated on the survey data set of all 34 participants to test the strength of association between changes in sitting time while at work and changes in back pain, office behaviors, and mood states. All data were analyzed by using SAS software (SAS Institute, Inc, Cary, North Carolina).

## Outcome

The participation rate in the Take-a-Stand Project was 62%. At baseline, participants perceived their health as very good to excellent, were physically active, reported very good or excellent levels of estimated cardiorespiratory fitness (18), were normal weight, and did not report any major limitations on work performance because of physical or emotional health concerns (Table 1).

**Table 1:** Participant Characteristics by Group Measured by Survey at Baseline, the Take-a-Stand Project, Minneapolis, Minnesota, 2011

Characteristic	Intervention Group (n = 24), Mean (SD)	Comparison Group (n = 10), Mean (SD)
Age, y	38.4 (11.4)	44.2 (11.9)
Female sex, %	96 (0.2)	80 (0.4)
Body weight, lb	138.1 (22.1)	143 (20.2)
Body mass index, kg/m^2^	22.8 (2.6)	22.8 (2.2)
Physical activity, percentage meeting guidelines**^a^**	75 (0.4)	70 (0.5)
Cardiorespiratory fitness (estimated maximal oxygen consumption, mL/kg/min**^b^**)	35.6 (6.6)	34.0 (8.0)
**Self-perceived general health status, %**		
Excellent	42 (0.5)	40 (0.5)
Very good	37 (0.5)	60 (0.5)
Good	21 (0.4)	NA
Fair	NA	NA
Poor	NA	NA
**Role physical^c^, %**		
Extremely	NA	NA
Quite a bit	NA	NA
Moderately	8 (0.3)	NA
A little bit	8 (0.3)	NA
Not at all	84 (0.4)	100
**Role emotional^d^, %**		
Extremely	NA	NA
Quite a bit	NA	10 (0.3)
Moderately	17 (0.4)	NA
A little bit	21 (0.4)	60 (0.5)
Not at all	62 (0.5)	30 (0.5)
**Job types, %**		
Administrative support	8.3 (0.3)	NA
Customer service	12.5 (0.3)	NA
Account management	12.5 (0.3)	10 (0.3)
Health coach	54.2 (0.5)	60 (0.5)
Manager/leader	4.2 (0.2)	20 (0.4)
Other	8.3 (0.3)	10 (0.3)

Abbreviation: SD, standard deviation; NA, not applicable.

^a^ Based on the 2008 US Department of Health and Human Services Physical Activity Guidelines (www.health.gov/paguidelines).

^b^ Estimated maximal oxygen consumption based on self report as per the prediction equation by Ainsworth, et al (18). Cardiorespiratory fitness categories are based on Heyward, p 48 (21).

^c^ The degree of problems with work or other daily activities as a result of physical health issues.

^d^ The degree of problems with work or other daily activities as a result of emotional health issues.

The ESM data are depicted as the average score for each period by group (Figure 2). For the comparison group, average ESM scores comparing period 1 with period 2 (*P* = .65), period 1 with period 3 (*P* = .12), and period 2 with period 3 (*P* = .25) did not show any significant differences ( > .05 and *P* > .017). The intervention group, however, increased average ESM scores (representing reduced sitting behavior) significantly, by 224% during period 2 compared with period 1 (*P* < .001) and reduced average ESM scores (representing increased sitting behavior) during period 3 compared with period 2 (*P* < .001) to significantly below baseline levels (*P* = .002).

**Figure:**
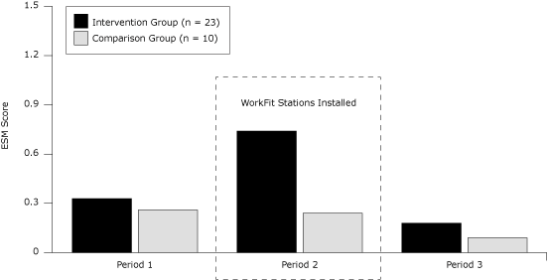
Figure 2. Average experience-sampling methodology (ESM) score by group and period of the study, the Take-a-Stand Project, Minneapolis, Minnesota, 2011. ESM score represents the average score by period generated by participants responding to the questions whether they were sitting (0), standing (1), or walking (2), respectively. Period 1 represents the baseline period (average across 1 week), Period 2 represents the intervention period (average across 4 weeks), and period 3 represents the postintervention period (average across 2 weeks).

**Table:** 

Period	Intervention Group (n = 23)	Comparison Group (n = 10)
**Period 1**	0.33	0.26
**Period 2**	0.74	0.24
**Period 3**	0.18	0.09

We conducted surveys at baseline (T1), at the end of period 2 (T2), and at the end of period 3 (T3) (Table 2). Sitting time while at work declined in the intervention group from T1 to T2 by 66 minutes per day (*P* = .03) and increased back to baseline levels following removal of the sit-stand devices from T2 to T3 (*P* = .001). The comparison group increased sitting time across all periods such that sitting time at T2 was 17 minutes per day longer compared with T1 (*P* = .31) and increased another 21 minutes from T2 to T3 (*P* = .03) such that the final recording at T3 was 38 minutes longer than at T1 (*P* = .04).

**Table 2:** Changes in Sitting Time, Back Pain, Office Behavior, and Profile of Mood States for Study Groups, Measured by Survey at Baseline (T1), End of Week 5 (T2), and End of Week 7 (T3), the Take-a-Stand Project, Minneapolis, Minnesota, 2011^a^

Variable	Intervention Group (n = 23			Comparison Group (n = 10)		
	T1–T2 (*P*)	T1–T3 (*P*)	T2–T3 (*P*)	T1–T2 (*P*)	T1–T3 (*P*)	T2–T3 (*P*)
**Sitting time, back pain, and office behavior**						
Time spent sitting at work, min/d	409–343 (.03)	409–417 (.57)	343–417 (.009)	441–458 (.31)	441–479 (.04)	458–479 (.03)
Low back pain (0-10 scale) ^b^	1.04–0.87 (.64)	1.04–1.52 (.21)	0.87–1.52 (.07)	1.1–1.3 (.51)	1.1–1.3 (.64)	1.3–1.3 (>.99)
Upper back and neck pain (0-10 scale) ^b^	2.65–1.43 (.008)	2.65–2.26 (.26)	1.43–2.26 (.03)	2.4–2.0 (.10)	2.4–2.3 (.59)	2.0–2.3 (.19)
Time spent in face-to-face office conversations, % of work day	7.0–8.1 (.50)	7.0–5.6 (.27)	8.1–5.6 (.19)	6.7–6.2 (.62)	6.7–10.5 (.09)	6.2–10.5 (.07)
Time spent in physical activity, % of work day	2.6–2.3 (.63)	2.6–3.2 (.55)	2.3–3.3 (.35)	4.5–2.6 (.37)	4.5–3.0 (.61)	2.6–3.0 (.28)
**Mood states**						
Fatigue (0–20 scale)^c^	6.5–4.8 (.01)	6.5–6.1 (.61)	4.8–6.1 (.08)	4.9–5.6 (.38)	4.9–6.7 (.11)	5.6–6.7 (.10)
Anger (0–28 scale)^c^	4.2–3.2 (.17)	4.2–3.6 (.26)	3.2–3.6 (.56)	3.5–3.2 (.82)	3.5–2.9 (.66)	3.2–2.9 (.69)
Vigor (0–24 scale)^c^	11.9–13.8 (.02)	11.9–10.4 (.14)	13.8–10.4 (<.001)	11.3–13.2 (.16)	11.3–12.3 (.57)	13.2–12.3 (.21)
Tension (0–24 scale)^c^	5.5–4.0 (.048)	5.5–4.1 (.005)	4.0–4.1 (.84)	4.6–4.6 (>.99)	4.6–4.9 (.70)	4.6–4.9 (.63)
Self-esteem (0–12 scale)^c^	6.5–6.9 (.42)	6.5–5.4 (.09)	6.9–5.4 (.03)	7.8–7.9 (.86)	7.8–7.1 (.35)	7.9–7.1 (.27)
Confusion (0–20 scale)^c^	4.8–3.8 (.03)	4.8–3.7 (.007)	3.8–3.7 (.59)	4.9–5.5 (.24)	4.9–4.9 (1.0)	5.5–4.9 (.37)
Depression (0–24 scale)^c^	2.0–1.3 (.04)	2.0–1.9 (.74)	1.3–1.9 (.20)	1.4–1.2 (.76)	1.4–2.1 (.39)	1.2–2.1 (.23)
Total mood disturbance (64–180 scale)^c^	104.7–96.3 (.003)	104.7–103.6 (.69)	96.3–103.6 (.04)	100.2–99.0 (.74)	100.2–102.1 (.69)	99.0–102.1 (.28)

^a^ *P* values presented are calculated based on paired *t* tests and adjusted by using the Bonferroni multiple comparison correction method and may be interpreted at *P* < .05 and *P* < .017.

^b^ Denotes severity of pain from 0 (no discomfort) to 10 (extremely uncomfortable).

^c^ Denotes the scale range for domain-specific Profile of Mood States questionnaire scales.

Upper back and neck pain declined significantly from T1 to T2 for the intervention group (*P* = .008). This improvement was negated during period 3 (T2 to T3; *P* = .027). No significant changes were observed for either the intervention or the comparison group for lower back pain, time spent in face-to-face interactions with coworkers, or time spent in physical activity breaks.

The intervention group experienced significant improvements in self-reported mood states. Significant improvements from T1 to T2 were noted for fatigue (*P* < .017), vigor (*P* = .017), tension (*P* < .05), confusion (*P* < .05), depression (*P* < .05), and total mood disturbance (*P* < .017). Following removal of the sit-stand devices at T2, vigor and total mood disturbance returned to baseline levels (*P* < .05). Following a slight improvement during the intervention period, self-esteem decreased after removal of the devices (*P* < .05) to below baseline levels. No significant changes in any of the mood states were noted at any of the measurement points for the comparison group.

Pearson’s correlation coefficients were calculated for all participants to test the association between changes during period 1 and 2 of the project in sitting time while at work and changes in back pain, office behaviors, and mood states. Reductions in sitting time were significantly associated with reductions in upper back and neck pain (*r* = 0.47; *P* = .006), fatigue (*r* = 0.44; *P* = .01), confusion (*r* = 0.46; *P* = .007), and total mood disturbance (*r* = 0.35; *P* = .046).

At the end of week 7, the intervention group was asked several questions about specific benefits of alternating between a seated and standing position. Results indicated that 87% felt more comfortable, 87% felt energized, 75% felt healthier, 71% felt more focused, 66% felt more productive, 62% felt happier, and 33% felt less stressed as a result of having the sit-stand device installed at their work stations.

Finally, the open field comments provided by the participants as part of the survey conducted at week 7 generally supported the observations made by the quantitative analyses. Comments included observations related to less low back pain and shoulder tension, posture improvement, decreased wrist and elbow pain, and increased comfort. No negative comments were received.

## Interpretation

The Take-a-Stand Project was designed to reduce prolonged sitting time at work and improve selected health outcomes for employees with sedentary jobs. The installation of a sit-stand device in the context of a corporate health and well-being program (10) was effective at increasing nonsitting time, reducing upper back and neck pain, and improving mood states. Furthermore, the removal of the sit-stand device largely negated all observed improvements within a 2- week period.

The Take-a-Stand Project was successful at increasing nonsitting behavior by 224% based on ESM and by 66 minutes per day (ie, a 16.1% reduction in sedentary time) based on survey responses. A recent systematic review noted that while reducing sitting time is emerging as a new work place health priority, evidence is lacking to show that work place interventions for reducing sitting are effective (14). In a recent study, following a week of monitoring to measure baseline activity and sitting behavior, height-adjustable desks were installed in the work spaces of 11 participants (13). Although participants were encouraged to use the work stations during the second week of monitoring, the authors failed to document an overall reduction in sitting time. Although studies have attempted to increase physical activity through the use of office-based devices such as treadmill workstations (11), height-adjustable desks (13), or place steppers (20), most of these reports have focused on reductions in sitting time as a secondary outcome. Few studies report health benefits resulting from successful interventions to reduce sitting time.

Our study succeeded in changing the physical environment of employees by introducing a sit-stand device and in recording a significant reduction in sitting time during work. Reductions in sitting time correlated significantly with improved outcomes for upper back and neck pain as well as various mood states. This level of intervention effect warrants further study using more rigorous experimental study design criteria.

Several limitations of the Take-a-Stand Project should be noted. First, the project did not allow for randomization in study group assignment; thus, we cannot assume causality. In addition, a certain degree of bias needs to be acknowledged, because all data considered in the project were based on self-report. The Take-a-Stand Project was conducted among a small number of participants, which may have limited our ability to apply scientific rigor to statistical analyses. Finally, the project was conducted among health-conscious, physically active, aerobically fit, and generally healthy participants. On the one hand, they may have been more likely as a group to respond to the intervention. On the other hand, they may have been less likely to experience an intervention effect on health outcomes because there was little room for improvement (eg, baseline low back pain ratings were slightly above 1 on a 10-point scale).

Our study’s limitations need to be balanced against several strengths. First, the study design included a comparison group and used an interrupted time series to optimize the likelihood that the results are not due to chance. Second, the use of ESM brings an additional degree of confidence that the observed improvements in sedentary (prolonged sitting) behavior are real effects. Finally, this project was conducted in a real-world, practice-based setting with high generalizability, and it generated data that may be used to support decision-making regarding resource investments for worker health and well-being programs.

On the basis of the results, additional research seems warranted. The data we describe support the need for additional research into innovative methods that reduce prolonged sitting time during the work day and consider outcomes in the domains of health protection (eg, ergonomics and injury prevention), health promotion (eg, health behaviors, psychosocial factors, disease prevention), and productivity (eg, absenteeism, presenteeism). Furthermore, interventions that influence various aspects of the work environment (eg, physical, psychosocial) and focus on an integrated approach to worker health protection and promotion, such as the Take-a-Stand Project, may have benefits that exceed those gained by initiatives focused on only 1 approach.

The Take-a-Stand Project was able to show that improvements in health factors such as back health indicators and mood states are directly related to reductions in sitting time of workers engaged in sedentary job tasks. Overall, this project was successful in reducing sedentary behaviors of workers and suggests reduced sitting time improves worker health.

## Appendix. Survey Questionnaire Administered to Take-a-Stand Project Participants, Minneapolis, Minnesota, 2011

This appendix is available for download as a Microsoft Word file [DOC - 57KB].
