# Supplementary material for: Reducing Occupational Sitting Time and Improving Worker Health: The Take-a-Stand Project, 2011
Source: Prev Chronic Dis. 2012 Oct 11;9:E154. doi: 10.5888/pcd9.110323 (PMC3477898; doi:10.5888/pcd9.110323)
Supplement: Supplementary file 1 [file 11_0323_01.doc]

**Appendix Survey Questionnaire Administered to Take-a-Stand Project Participants, Minneapolis, Minnesota, 2011**

**Demographics**

| How old are you? *(1^st^ questionnaire only)* | [drop down] |
| --- | --- |
| What is your gender? *(1^st^ questionnaire only)* | 🔿 Male  🔿 Female |
| How tall are you? *(1^st^ questionnaire only)* | __________ feet, __________ inches  (round to the nearest inch) |
| How much do you weigh? *(all 3 questionnaires)* | __________ pounds |

Physical Activity

| How many ***days*** in a usual week do you do ***vigorous activities*** for at least 10 minutes at a time, such as running, aerobics, heavy yard work, or anything else that causes a large increase in your breathing or heart rate? | 🔿 0 🔿 4  🔿 1 🔿 5  🔿 2 🔿 6  🔿 3 🔿 7 |
| --- | --- |
| On days when you do ***vigorous activities*** for at least 10 minutes at a time, how much ***total time (minutes)*** each day do you spend doing these activities? | [drop down menu listing numbers from 10 through 120 and 120 minutes or more]  🔿 I do not do vigorous activities for at least 10 minutes at a time |
| How many ***days*** in a usual week do you do ***moderate activities*** for at least 10 minutes at a time, such as brisk walking, bicycling, vacuuming, gardening, or anything else that causes a small increase in your breathing or heart rate? | 🔿 0 🔿 4  🔿 1 🔿 5  🔿 2 🔿 6  🔿 3 🔿 7 |
| On days when you do ***moderate activities*** for at least 10 minutes at a time, how much ***total time (minutes)*** each day do you spend doing these activities? | [drop down 10 thru 120; 120 minutes or more]  🔿 I do not do moderate activities for at least 10 minutes at a time |
| Considering a 7-day period (a week), how many times on average do you do strenuous exercise (heart beats rapidly) for more than 15 minutes during your free time? | [drop-down menu listing numbers from 0 through 20 or more] |

Sitting

| In a usual day, how much time do you sit at work? | __________ hours____ ___minutes |
| --- | --- |

General Health Status

| In general, would you say your health is: | 🔿 Excellent  🔿 Very Good  🔿 Good  🔿 Fair  🔿 Poor |
| --- | --- |
| In the last month, how much difficulty did you have doing your work or other regular activities as a result of your physical health? | 🔿 Not at all  🔿 A little bit  🔿 Moderately  🔿Quite a bit  🔿 Extremely |
| In the last month, to what extent have you accomplished less than you would like in your work or other daily activities as a result of your emotional health such as feeling stressed, depressed or anxious? | 🔿 Not at all  🔿 A little bit  🔿 Moderately  🔿Quite a bit  🔿 Extremely |

Pain

| On a scale of 0-10, please rate your current level of: | 🔿 0 = No discomfort  🔿 1  🔿 2  🔿 3  🔿 4  🔿 5  🔿 6  🔿 7  🔿 8  🔿 9  🔿 10 = Extremely uncomfortable |
| --- | --- |
| Lower back pain or discomfort | *[Use scale above]* |
| Upper back, neck and shoulder pain | *[Use scale above]* |

Office Behavior

| What proportion of the time do you conduct your work within the Health Promotion Department HPD using the following methods |  |  |
| --- | --- | --- |
|  | Informal face to face conversations | _____% |
|  | Moving breaks | _____% |

Feelings (POMS Questionnaire)

| The following list describes feelings people have. Please read each one carefully and select the answer that best describes your feelings in the past week. | 🔿 Not at all  🔿 A little  🔿 Moderately  🔿 Quite a bit  🔿 Extremely |
| --- | --- |
| Worn out | *[Use scale above for all feelings]* |
| Peeved |  |
| Cheerful |  |
| Restless |  |
| Embarrassed |  |
| Bewildered |  |
| Hopeless |  |
| Weary |  |
| Bitter |  |
| Vigorous |  |
| Nervous |  |
| Ashamed |  |
| Forgetful |  |
| Helpless |  |
| Concentrate |  |
| Resentful |  |
| Full of pep |  |
| Miserable |  |
| Furious |  |
| Lively |  |
| On-edge |  |
| Proud |  |
| Confused |  |
| Sad |  |
| Fatigued |  |
| Grouchy |  |
| Active |  |
| Tense |  |
| Competent |  |
| Bushed |  |
| Worthless |  |
| Exhausted |  |
| Angry |  |
| Energetic |  |
| Uneasy |  |
| Satisfied |  |
| Uncertain |  |
| Anxious |  |
| Discouraged |  |
| Annoyed |  |

| What specific benefits did you gain by alternating between a seated and standing position: | *(check all that apply)*  🔿 Uncertain  🔿 Less stressed  🔿 More comfortable  🔿 More productive  🔿 More focused  🔿 Happier  🔿 Energized  🔿 Healthier  🔿 None of the above  🔿 Other (please specify):  __________________________________ |
| --- | --- |
